# Rat and Mouse Brain Tumor Models for Experimental Neuro-Oncology Research

**DOI:** 10.1093/jnen/nlac021

**Published:** 2022-04-21

**Authors:** Upasana Sahu, Rolf F Barth, Yoshihiro Otani, Ryan McCormack, Balveen Kaur

**Affiliations:** 1 From the Department of Neurosurgery, McGovern Medical School, University of Texas Health Science Center at Houston, Houston, Texas, USA; 2 Department of Pathology, The Ohio State University, Columbus, Ohio, USA

**Keywords:** 9L, C6, F98 rat brain tumor models, Genetically engineered and humanized mouse brain models

## Abstract

Rodent brain tumor models have been useful for developing effective therapies for glioblastomas (GBMs). In this review, we first discuss the 3 most commonly used rat brain tumor models, the C6, 9L, and F98 gliomas, which are all induced by repeated injections of nitrosourea to adult rats. The C6 glioma arose in an outbred Wistar rat and its potential to evoke an alloimmune response is a serious limitation. The 9L gliosarcoma arose in a Fischer rat and is strongly immunogenic, which must be taken into consideration when using it for therapy studies. The F98 glioma may be the best of the 3 but it does not fully recapitulate human GBMs because it is weakly immunogenic. Next, we discuss a number of mouse models. The first are human patient-derived xenograft gliomas in immunodeficient mice. These have failed to reproduce the tumor-host interactions and microenvironment of human GBMs. Genetically engineered mouse models recapitulate the molecular alterations of GBMs in an immunocompetent environment and “humanized” mouse models repopulate with human immune cells. While the latter are rarely isogenic, expensive to produce, and challenging to use, they represent an important advance. The advantages and limitations of each of these brain tumor models are discussed. This information will assist investigators in selecting the most appropriate model for the specific focus of their research.

## INTRODUCTION

Glioblastomas (GBMs) are the most common primary, high-grade, malignant brain tumor, constituting 14.5% of all brain tumors with an estimated >12 000 new patient diagnoses and an almost equal number of deaths occurring annually in the United States (1, 2). Despite advances in their surgical treatment, adjuvant chemo-radiotherapy using temozolomide (TMZ), and novel modalities such as alternating electric fields, the prognosis of GBMs remains dismal (3–8). Although molecular profiling of GBMs has uncovered many different, potential molecular targets, the clinical utility of these therapies has resulted in only modest increases in overall patient survival rates.

Rat and mouse brain tumor models have led to the development of a number of therapeutic approaches for the treatment of human GBMs and have been the most widely used in experimental neuro-oncology. Historically, the first GBM models were generated by administering nitrosourea to pregnant rats and mice in order to induce brain tumors in their progeny. The 3 most widely used of these rat models are the C6 glioma, the 9L gliosarcoma, and the F98 glioma. The RG2, BT4C, RT-2, and CNS-1 models have been used much less frequently; interested readers are referred to the previous review of Barth and Kaur for information relating to them (9). The present review focuses on the C6, 9L, and F98 rat brain tumor models and a larger number of mouse tumor models.

Until recently, mouse models were limited to the GL261, which has been used less frequently than the rat models (10). However, the ability to produce genetically engineered glioma cell lines has increased their use over the past 15 years (11). The relative advantages and disadvantages of the rat and mouse models are summarized in Table 1. It was first reported in the late 1960s that CNS tumors could be induced reproducibly and selectively in either adult rats or the progeny of pregnant females that had been given intravenous (i.v.) injections of N-methylnitrosourea (MNU) over several weeks, or a single dose of N-ethyl-N-nitrosourea (ENU). As summarized in Table 2, these studies led to the development of the C6, 9L, and F98 rat gliomas (12). Although widely used, patient-derived xenograft (PDX) models, based on the intracerebral (i.c.) implantation of human brain tumor cell lines into immunologically deficient mice, will be briefly discussed. However, more information relating to them is provided in the review by Candolfi et al (13).

**TABLE 1.** Advantages and Disadvantages of Rat Versus Mouse Brain Tumor Models

Advantages	Disadvantages
Larger size of the rat brain permits more precise stereotactic implantation and a longer time interval until death. Larger tumor size permits better in vivo localization by various imaging methods. Larger amounts of various therapeutic agents can be administered intracerebrally (i.c.), thereby permitting critical evaluation of their effectiveness. Currently more extensive literature on rat brain tumors compared to mouse tumors, which can be useful in developing new therapeutic modalities.	Rat brain tumor models frequently have not been genetically engineered to study roles of genetic factors, signaling pathways, tumor growth, and invasion. Few genetically engineered tumor cell lines are available. Fewer monoclonal antibodies directed against rat surface antigens and chemokines compared to those of the mouse. Rats are more expensive to purchase and maintain than mice.

**TABLE 2.** Comparison of the C6, 9L, and F98 Rat Brain Tumor Models

Tumor	Strain of origin	**Mode of tumor induction** *	Minimum i.c. innoculum	Immuno- genicity	Pattern of growth	Molecular markers
C6	Wistar	MNU	10^4^	strong	circumscribed	Mutant *p16/CDkn2a/ Ink4a;* no expression of *p16* and *p19ARF* mRNAs or of wildtype *p53*
9L	Fischer	MNU	10^4^	strong	circumscribed	Mutant *p53*, increased expression of *TGFα* and *EGFR*; decreased expression of *FGF-2, FGF-9, FGFR-1* and *PDGFRβ*
F98	Fischer	ENU	10^1^ – 10^2^	weak	infiltrative	Increased expression of *PDGFβ, Ras, EGFR, cyclin D1* and *cyclin D2*

* Abbreviations: *MNU*: methylnitrosourea; *ENU*: *N*-ethyl-*N*-nitrosurourea

The utility of these rat and mouse brain tumor models notwithstanding, none of them exactly simulates human high-grade gliomas. Rat brain tumor models, and to a much lesser extent, mouse models have provided very useful information for the development of a number of new treatment approaches for human high-grade gliomas. Recently, mouse brain tumor models have become especially useful, since they can be genetically manipulated in a variety of ways to better understand the effects of different mutations on their tumorgenicity and response to different therapeutic modalities (14). Useful brain tumor models should: (1) be derived from glial cells; (2) grow and be cloned in vitro and propagated in vivo; (3) have reproducible and predictable growth rates; (4) have glioma-like growth characteristics within the brain; (5) allow the host to survive for a sufficient period of time after i.c. tumor implantation for evaluation of therapeutic efficacy; and (6) be either non- or weakly immunogenic in order to eliminate host immune responses and thereby be more predictive of human GBMs. Stereotactic i.c. implantation using suspensions of tumor cells has become the standard for in vivo studies in rat brain tumor models (15). This has reduced spinal and extra-cranial dissemination, especially when combined with the use of plastic (16) or metallic screws with an entry port (17, 18), for the implantation of tumor cells. These can be left in place to facilitate subsequent administration of therapeutic agents.

Humanized immunocompetent mouse glioma models in mice have proven to be useful tools for investigating the therapeutic effects and interaction between tumor cells and the tumor microenvironment (TME). Immunocompetent mouse models that recapitulate the TME, including immune effector cells and vasculature, are important for evaluating immunomodulating therapies. The development of immunodeficient mice has led to the use of PDX in mice. While these tumors simulated the molecular background of GBMs, they failed to recapitulate the tumor-host interaction and the TME of brain tumors. Advances in molecular biology have led to the establishment of genetically engineered mouse (GEM) models that simulate the molecular alterations in GBMs in an immunocompetent environment. However, they impose restraints for exploring inherently species-specific biological therapies.

Similar to the rat models, chemically induced mouse glioma models are frequently used. Among these, the most frequently used is the GL261 glioma. This was produced by i.c. injection of methylcholanthrene (MCA), followed by serial i.c. and subcutaneous (s.c.) transplantation of tumor fragments (19). Intracerebral inoculation of GL261 cells reliably results in tumors expressing activating mutations in the Ras pathway along with loss of tumor suppressors such as p53; in this respect, they are identical to human GBMs. This model has been used extensively to study glioma stem cells and their response to therapies. The development of biological therapies such as oncolytic human herpes simplex virus (HSV) requires a human receptor for viral entry. GL261 cells, transduced with HSV and entering the cell via the receptor nectin 1 (GL261-N4), have been generated for the evaluation of HSV-based therapeutics (20). While this model is perhaps the most frequently utilized of the mouse models it is also considered to be immunogenic and thus unsuitable for any other type of therapy studies. CT-2A is another mouse glioma cell line that was developed by chemical induction with MCA (21). These tumors are deficient in PTEN and have a dysregulated PI3K pathway which made them useful to evaluate PTEN and/or PI3K signaling effects (22).

## RAT C6 GLIOMA

### Origin

The number of citations in Google Scholar (over 16 400 as of February 2022) shows that the C6 rat glioma has been the most widely used of all rodent brain tumor models. It was produced by Benda et al (23) and Schmidek et al (24) in William Sweet’s laboratory in the Department of Neurosurgery at the Massachusetts General Hospital by repeatedly administering MNU to outbred Wistar rats over 8 months. Following the development of neurological signs, the rats were killed and their tumors were excised and processed for tissue culture. One of these tumors, designated “#6,” subsequently was cloned and shown to produce S-100 protein and renamed the C6 glioma (23, 25). It was originally classified as an astrocytoma, but, following accession to the American Type Culture Collection (ATCC), Rockville, MD (ATCC# CCL-107), it was reclassified as a glial tumor. In addition, 2 C6 sublines with the LacZ reporter gene are currently available from the ATCC (C6/lacZ 7 [CRL-2303] and C6/lacZ [CRL-2199]).

### Genomics

As summarized in Table 2, genes expressed by the C6 glioma relevant to human GBMs include *Cdkn2a*, *Cdkn2b*, *Pik3ca*, and *Cdkn2a* and *b* with wild-type p53 mutations and mutant *p16/Cdkn2a/Ink4a* locus (26), with no expression of *p16* and *p19ARF* mRNAs (27). The oncogene *Pik3ca* matched the Catalogues of Somatic Mutations in Cancer (COSMIC) database with the amino acid C90V substitution and location of Grade IV astrocytomas (28). As summarized in Table 2, molecular characterization comparing changes in gene expression between the C6 glioma and rat stem cell-derived astrocytes, revealed that gene expression in the C6 tumor resembled those reported in human gliomas (26). It also had increased expression of the *PDGFβ*, *IGF-1*, *EGFR*, and *Erb3/Her3* genes, which frequently are overexpressed in human gliomas compared to non-neoplastic astrocytes (27, 29, 30). The significance of *PDGF* in gliomagenesis was established in adult rats by transfecting white matter with a retrovirus encoding *PDGF* and green fluorescent protein. All of the animals developed tumors, derived from both uninfected and infected glial progenitors, suggesting that *PDGF* was involved in both autocrine and paracrine stimulation of glial progenitor cells (31). There was reduced expression of IGF-2, FGF-9, and FGF-10 relative to astrocytes, but overexpression of IGF-1. Expression of *Ras* and *Ras* guanine triphosphate activator protein was increased, which was similar to the increased activity of the *Ras* pathway observed in human gliomas (26, 32). In contrast, to human GBMs, there was increased expression of the *Rb* gene (29). A stably expressing ß-galactosidase clone of C6 cells (ATCC; #CRL-2303) has also been described (33); this has permitted immunohistochemical analysis of these tumors. However, the ß-galactosidase marker protein can serve as a tumor antigen, and immunization of rats against the reporter gene protected the animals against tumor growth (33).

### Experimental Studies

As recently summarized by Giakoumettis et al (34), the C6 rat glioma model has been widely used in experimental neuro-oncology to evaluate the efficacy of a variety of therapeutic modalities, including chemotherapy (35), anti-angiogenic therapy (36), proteosome inhibitors (37), treatment with toxins (38), radiation therapy (39), photodynamic therapy (40), oncolytic viral therapy (41), and gene therapy (42). However, since this tumor arose in an outbred Wistar rat, there is no syngeneic host in which it can be propagated and it cannot be considered as the gold standard of rat brain tumors (34). The C6 glioma is immunogenic in Wistar and BDX rats even with a small i.c. inoculum of tumor cells (43). Therefore, it is not suitable for evaluating the efficacy of immunotherapy since human high-grade gliomas are of indeterminant immunogenicity. This in part may be due to the immunosuppressive TME of GBMs, the molecular and cellular heterogeneity of these tumors (44), and T-cell dysfunction and exhaustion (45). This problem is exemplified by studies in which C6 glioma cells were transfected with an antisense cDNA expression vector that downregulated the constitutive production of IGF-1 (45, 46). Not recognizing that the tumor was of Wistar origin, the authors, unfortunately, used BD IX rats, which they thought was the strain of origin due to some ambiguity in the literature. Subsequently, they reported that BD IX rats, which had been immunized with the C6 anti-sense IGF-1 transfected cells, were resistant to both s.c. and i.c. challenge of the C6 glioma. Similarly, Wistar rats bearing C6 gliomas (s.c. or i.c.) developed potent humoral and cellular immune responses to the tumor, and rats challenged simultaneously with s.c. and i.c. tumors, had a survival rate of 100% (43). However, this could be attributed to an alloimmune response.

### Genetically Engineered EGFR-Expressing C6 Gliomas

The discovery of EGFR gene amplification in GBMs led to the development of a genetically engineered subline C6 glioma which expressed amplified human EGFR and was designated C-EGFR (47). Rats that received i.c. implants of 10^3^ or 10^4^ cells had survival times that were nearly identical to those of animals implanted with wild-type C6 cells. Unexpectedly, however, 33% of the rats that were injected with 10^5^ or 10^6^ C6-EGFR cells survived longer (750 days), compared to 27 days for those injected with 10^5^ or 10^6^ wild-type cells. This suggested that the long-term surviving animals had developed a xeno-immune response directed against human EGFR and that an inoculum of 10^3^ or 10^4^ cells was insufficient to evoke an immune response.

### Immune Response to the C6 Glioma

Gieryng et al (48) used flow cytometry to characterize the immune environment of C6 gliomas that had been implanted i.c. into outbred Wistar rats. They found that there was a significant increase in pro-tumorigenic microglial/macrophages, T helper (Th), T regulatory cells (Treg), and rare infiltrating cytotoxic T cells (Tc). Transcriptomic analysis of the tumor-bearing cerebral hemispheres revealed overexpression of invasion- and immunosuppression-related genes, consistent with an immunosuppressive TME. They concluded that the accumulation of Th and Treg cells, combined with reduced numbers of Tc could result in weakened anti-tumor responses. However, since this study was carried out in outbred Wistar rats, the strain of origin of the C6 glioma, the relevance of these findings to studies carried out in a syngeneic model such as the 9L gliosarcoma and the F98 glioma is questionable, despite the significant time and effort that was devoted to this research.

Because C6 glioma cells are allogeneic in all inbred rat strains, this characteristic should provide a strong cautionary note for studies employing this brain tumor model for any therapy studies. Despite this limitation, the C6 glioma model continues to be used in a wide variety of studies related to brain tumor biology (36), including studies on tumor growth, invasion, migration, blood-brain barrier (BBB) disruption, neovascularization, growth factor regulation and production, and biochemical studies (49–51), although it is not the “gold standard” for such research (34).

## RAT 9L GLIOSARCOMA

### Origin

**FIGURE 1.**
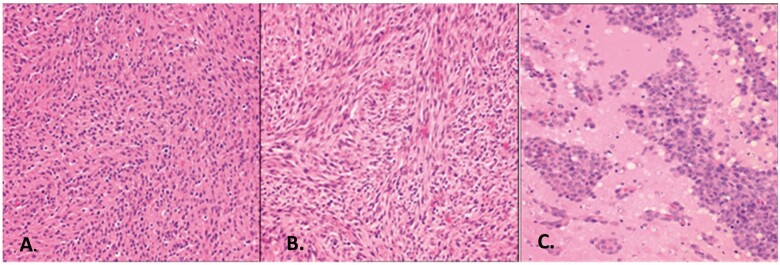
Histopathologic features of the C6, 9L, and F98 brain tumors. **(A)** The C6 glioma is composed of a pleomorphic population of cells with nuclei ranging from round to oblong. A herring-bone pattern of growth is seen in some areas and there is focal invasion of contiguous normal brain. There are scattered foci of necrosis with pseudo-palisading of tumor cells at the periphery. **(B)** The 9L gliosarcoma is composed of spindle-shaped cells with a sarcomatoid appearance. A whorled pattern of growth is seen with sharp delineation of the margins of the tumor with little invasion of contiguous normal brain. **(C)** The F98 glioma is composed of a mixed population of spindle-shaped cells with fusiform nuclei, frequently forming a whorled pattern of growth, and a smaller subpopulation of polygonal cells with round to oval nuclei. There is extensive invasion of contiguous normal brain with islands of tumor cells at varying distances from the main tumor mass, which form perivascular clusters. Usually, there is a central area of necrosis filled with tumor cell ghosts.

A search of Google Scholar revealed that the 9L gliosarcoma has been the second most widely used experimental rat brain tumor model, with over 6770 Google Scholar citations (February 2022). It was produced by the i.v. injection of 5 mg/kg of MNU to Fischer rats for 26 weeks (24, 52). It originally was designated as “tumor #9,” which subsequently was cloned at the Brain Tumor Research Center, University of California, San Francisco, and then was designated “9L” (24, 52, 53) and “T9” by Denlinger et al (54). The tumor cells could be propagated in tissue culture*,* which made them very useful for both in vitro and in vivo studies of the effects of various therapeutic agents on brain tumors. 9L cells can be implanted i.c. into syngeneic Fischer rats, after which they develop into rapidly growing tumors that are composed of spindle-shaped cells with a sarcomatoid appearance (see Figure 1B). The tumor margins are sharply delineated with little obvious invasion into the contiguous normal brain (9).

### Genomics

As summarized in Table 2, the 9L gliosarcoma has a mutant *p53* gene (27) and normal expression of *p16* and *p19ARF* mRNAs, indicating that there is a wild-type *p16/Cdkn2a/INK4α* locus (26). Relative to rat cell-derived astrocytes, molecular characterization of the 9L revealed a significant increase in the expression of the genes encoding *TGFα* and its receptor and *EGFR* and decreased expression of *FGF-2*, *FGF-9*, and *FGFR-1* and *PDGFRβ* (29). Cancer stem-like cells have been demonstrated in the 9L cell line that grew as neurospheres in chemically defined medium and expressed the neural stem cell markers *Nestin* and *Sox2*. In vitro, they are self-renewable and differentiate into neuron- and glial-like cells (55). The neurospheres have a lower proliferation rate, express several anti-apoptotic and drug-related genes, and form tumors that are more aggressive than the parental 9L tumor (55).

### Experimental Studies

The 9L gliosarcoma model has been used widely to investigate the transport of drugs across the BBB and blood-tumor barriers (56–59), the mechanisms and development of drug resistance (60, 61), in magnetic resonance imaging (MRI) studies and positron emission tomography studies to evaluate tumor metabolism and hypoxia (62, 63), and in pharmacokinetic studies of bis-chlorethyl (59). Therapeutically, the 9L tumor has been used to study the effects of anti-angiogenic agents (64, 65), X-radiation (66), boron neutron capture therapy (BNCT) (67), chemotherapy (68, 69), gene therapy (70–73), immunotoxin treatment (74), oncolytic viral therapy (75, 76), and immunotherapy and cytokine therapy (71, 77). The 9L tumor also has been used following treatment to study the effect of BBB disruption (56), implantation of devices for repeated intratumoral drug delivery (18), and imaging (78).

### Immunogenicity of the 9L Gliosarcoma

Although impressive therapeutic results, including apparent cures of tumor-bearing animals, have been obtained, this tumor is highly immunogenic. It was first reported that rats immunized with X-irradiated 9L cells rejected both s.c. and i.c. tumor challenges, compared to 100% tumor takes in non-immunized rats (79). Unfortunately, this report first was published in the proceedings of a meeting and did not receive wide circulation, but other studies have confirmed its immunogenicity (54, 80). Transfection of 9L cells with the *s-Myc* gene under the control of a cytomegalovirus promotor resulted in complete suppression of tumor growth, and these animals subsequently rejected i.c. challenges with 9L cells. Histopathologic examination of the brains of these animals revealed heavy infiltrates of mononuclear cells and CD8-positive T lymphocytes, which suggested that tumor rejection was mediated by a potent T-cell anti-tumor immune response. Other more recent studies confirmed these observations. It has been reported that in vivo bystander cell killing (81, 82) observed following delivery of the HSV thymidine kinase gene (HSV-TK) (76, 83) and treatment with ganciclovir, was partially attributable to an anti-tumor immune response. The highly immunogenic nature of the 9L glioma must be kept in mind when using this model to evaluate the efficacy of novel therapeutic agents. Early studies employing radiation or chemotherapy alone were largely unsuccessful in curing the 9L tumor. However, the success obtained using BNCT (84–86), and gene therapy (87, 88), which is the result of an effective host response to the tumor, emphasizes the importance of anti-host-directed immune responses (73, 89). As reported by Smilowitz et al (84), 9L glioma-bearing rats that were treated by BNCT and received s.c. injections of irradiated 9L cells had prolonged survival times. This was attributed to immune response directed against the i.c. implants and against the tumor.

### Brainstem Tumor Model

Finally, the 9L gliosarcoma also has been used to develop a model for brainstem tumors (90). Progression to hemiparesis with the onset of symptoms occurred 17 days post-implantation of 9L cells into the brainstem. This model also has been used to evaluate the efficacy of convection enhanced delivery of carboplatin to the brainstem (90), and to study the response of recurrent, chemo-resistant gliomas. Two *bis*-chloroethyl nitrosourea (BCNU)-resistant cell lines were derived from 9L cells by treating them with BCNU in vitro or in vivo. Both of these cell lines formed tumors in 100% of the animals following i.c. implantation, and were much more invasive than the parental 9L cells (91) but, as previously mentioned, caution must be used in evaluating results obtained with this highly immunogenic tumor.

## F98 GLIOMA

### Origin and Genomics

The third most widely used brain tumor model, the F98 glioma (ATCC #CRL-2397), had over 4090 citations (Google Scholar, February 2022). It was produced in Adelbert Koestner’s laboratory at The Ohio State University by the i.v. administration of ENU (50 mg/kg body weight) to a pregnant Fischer 344 rat on the 20th day of gestation. Subsequently, the in vitro growth and morphology of the F98 glioma was described in detail leading to its classification based in its histopathology as an anaplastic or undifferentiated glioma (92). It is composed of a mixed population of spindle-shaped cells, the majority of which have fusiform nuclei, and a smaller number of polygonal cells with round to oval nuclei. There is extensive invasion of contiguous normal brain with islands of tumor cells at varying distances from the tumor mass, many of which form perivascular clusters (9, 16). Usually, there is a necrotic core, scattered mitotic cells, and non-glomeruloid neovascular proliferation (93). The tumor is GFAP- and vimentin-positive with minimal positivity for CD3-positive T cells (93). Similar to human GBMs, and summarized in Table 2, it overexpresses *Ras*, *PDGFβ*, and *cyclin D1*, *cyclin D2*, and *EGFR* and *Rb* relative to rat astrocytes (29) and has low expression of *BRCA1* (40, 69). It simulates human GBMs in a number of important ways, including its highly invasive pattern of growth (Figure 1) and weak immunogenicity, which have made the F98 particularly useful for the evaluation of the efficacy of a variety of therapeutic agents. It is refractory to a number of therapeutic modalities including systemic chemotherapy with carboplatin and paclitaxel, and it is poorly responsive to photon irradiation alone (94). This may be related in part to its functionally impaired *BRCA1* status that can favor genomic instability and impaired DNA repair (69). However, it is responsive to BNCT (95) and is highly responsive to a combination of 6 MV radiation in combination with carboplatin or cisplatin, administered i.c. by convection enhanced delivery (94). The F98 glioma model has also been used by Barth et al (96) to evaluate the efficacy of radio-iodine therapy, iodine-enhanced synchrotron stereotactic radiotherapy (47, 97), non-invasive MRI to visualize tumor growth (98), diffusion tensor imaging (99), tumor angiogenesis (100), molecular targeting of EGFR (101), and a variety of chemotherapeutic agents, including Nitrone OKN-007 (102), tonabersat, liposomal formulations of carboplatin (103), and TMZ.

### Immunogenicity of the F98 Glioma

Although the F98 glioma is less immunogenic than either the C6 or 9L rat gliomas, it is not non-immunogenic. Extensive studies to evaluate its immunogenicity were first carried out by Tzeng et al (104–106) over a 4-year period from 1989 to 1993. These in vitro and in vivo studies established that a subpopulation of lymphokine-activated killer cells had potent cytotoxic and cytostatic activity directed against F98 glioma cells (106). When they were injected i.c. together with tumor cells into Fischer rats, they produced a significant prolongation of mean survival time (MST; 46.1 days vs 22.3 days, p < 0.001), but all of the tumor-bearing animals eventually died. Next, a series of experiments were carried out to determine if s.c. implantation of F98 cells, followed by excision of the tumors and once weekly s.c. injection of irradiated F98 cells over 6 weeks would result in increased survival of these rats following i.c. implantation of 20 000 F98 cells. The MST of the immunized rats was increased by 4 days (22 vs 18.4) and, although this was statistically significant (p = 0.03), it was not biologically significant. Based on these and other studies, it was concluded that the F98 glioma was weakly immunogenic (104–106). This finding was further confirmed by transfection with the gene encoding B7.1 co-stimulatory molecule (107) or syngeneic cellular vaccination combination with GM-CSF (108), which did not enhance its immunogenicity. To the best of our knowledge, no other studies were carried out to evaluate the immunogenicity of the F98 glioma until 10 years later when Volovitz et al (109) reported that, although s.c. implants of the F98 glioma initially grew in Fischer rats, they rapidly regressed. Volovitz attributed this to an immune response, rather than an alternative explanation that the TME of s.c. implanted cells were not supportive of their growth. When these animals were challenged with an i.c. inoculum of 50,000 F98 glioma cells, a significant increase in MST was observed. Based on these results, Volovitz et al concluded that there was “split immunity” in that the s.c. implanted tumors regressed, and that those animals that had received i.c. injections had increased survival times, although they all died by 45 days. The essence of the studies of Tzeng et al (104–106) was that the F98 glioma was weakly immunogenic and Volovitz et al (109) concluded it was moderately immunogenic.

### Therapy Studies

The F98 glioma also has been used to study the molecular genetic alterations in GBMs (110), effects of infusion rates on drug distribution in i.c. chemotherapy with the nitrone compound OKN-007 tumors (102), and for suicide gene therapy with HSV-TK (111). F98 cells also have been injected into the pontine tegmentum of the brainstem of Fischer rats to produce a model for brainstem tumors (90). The radiobiological and histopathological characteristics of these tumors were comparable to aggressive, primary human brainstem tumors, which could facilitate preclinical testing of therapeutics to treat these lethal tumors. F98 cells have been stably transfected with expression vectors encoding for wild-type EGFR and EGFRvIII (101), and the resulting cell lines have been designated F98EGFR (ATCC# CRL-2948) and F98npEGFRvIII (ATCC# CRL-2949). Each expressed ∼10^5^ non-functional (i.e. non-phosphorylatable) receptor sites per cell, which was below the threshold number of 10^6^ sites per cell that can evoke a xeno-immune response against human EGFR in Fischer rats. These cell lines have been used in Fischer rats for studies on molecular targeting (112) to evaluate the therapeutic efficacy of boronated monoclonal antibodies (mAbs) and EGF for BNCT (101, 112, 113). The boronated mAb, L8A4, which is specific for EGFRvIII, and cetuximab, which recognizes wild-type EGFR, specifically targeted their respective receptor positive i.c. tumors after convection enhanced delivery and they were therapeutically effective following BNCT (101, 112, 113) and targeted delivery of methotrexate (114).

A bioluminescent, a transgenic luciferase expressing F98 cell line has been constructed by stably transfecting F98 cells with the luciferase gene (115). When implanted i.c. into the brains of Fischer rats, tumor size could be monitored by measuring its luminescence. This model permitted rapid, non-invasive imaging of i.c. tumor growth to evaluate novel therapeutic modalities (115). As with all rat brain tumor models, however, what may be therapeutically effective in the rat, may not be so in the human. However, it probably is safe to say that if a particular therapeutic approach is ineffective in a rat model, it is even more likely to be less so effective in humans.

## MOUSE BRAIN TUMOR MODELS

### Chemically Induced Mouse Brain Tumor Models: The GL261 GLIOMA

Immunocompetent mouse glioma models have proven to be useful tools for investigating the therapeutic effects and interaction between tumor cells and the TME. The need for immunocompetent animal models that recapitulate the TME, including immune effector cells and vasculature, are extremely important for evaluating immunomodulating therapies. Similar to the rat models, chemically induced mouse glioma models have been developed and are frequently used. Among these, the most frequently utilized glioma cell line is the GL261, which was developed by i.c. injection into C57BL/6 mice followed by serial i.c. and with MCA (19). Intracerebral inoculation of GL261 cells reliably results in tumors bearing activating mutations in the Ras pathway along with loss of tumor suppressors such as *p53*, which is identical to human GBM. The tumors are invasive but do not metastasize or spontaneously regress s.c. injection into syngeneic mice. Histologically, they resemble malignant ependymal tumors but display many features of human GBMs. They express *K-ras* and *p53* mutations that result in high expression of c-myc and major histocompatibility complex-1 (MHC-I) antigens and limited expression of MHC-II, B7-1, and B7-2 (116). They are, however, highly immunogenic in C57BL/6 mice. This model has been used extensively to study both glioma stem cells and their response to therapies. The recent development of biological therapies such as oncolytic human HSV requires a human receptor for viral entry. GL261 cells transduced with HSV and enter via the receptor nectin 1 (GL261-N4) have been generated for the evaluation of HSV-based therapeutics. This immunocompetent GBM model has been used to investigate the immunological effects associated with HSV-based therapeutics (20). While this model is the most frequently utilized mouse model for GBM, it is also considered to be immunogenic.

CT-2A is another mouse glioma cell line that was developed by chemical induction with MCA (21). The stem cell-like properties of these CT-2A tumor cells demonstrate a highly immune suppressive TME as reported by Khalsa et al (117); tumor cells are deficient in PTEN and have a dysregulated PI3K pathway which makes them useful to evaluate PTEN and/or PI3K signaling effects (22). However, their stem cell-like properties are associated with a highly immunosuppressive TME in contrast to GL26, which is considered more immunogenic. The advantages and limitations of various mouse brain tumor models are summarized in Table 3.

**TABLE 3.** Summary of Various Mouse Tumor Models and Their Advantages and Limitations

Model	Host	Advantages	Limitations
GL261	Mouse	Can be used in immunocompetent mice Can be used to study GSCs Can be used to study immunological effects associated with HSV-based therapeutics	Highly immunogenic relative to human GBMs Underlying genetics are different than human GBMs Inconsistency between studies
CT-2A	Mouse	Can be used in immunocompetent mice Can be used to study GSCs and how to augment immunogenicity of CD133+ stem cells Suitable tumor model for GBM research focused on immunotherapy of brain tumors	Overall immuno-suppressive microenvironment
GEMMs	Mouse	Can provide new insight into underlying molecular events and pathways for GBM initiation and progression Can directly investigate the impact of underlying tumor genomics on treatment response Can model tumor-stroma interactions to study their contribution to malignancy More similar development to human GBMs Can serve as an excellent tool to dissect the minimum genetic alterations necessary for malignant transformation	Lack of intra-tumoral heterogeneity Variability in tumor formation thereby limiting the use of precise treatment modalities Can be slow or inconsistent to grow tumors Can be expensive
SMA	Mouse	Can be used in immunocompetent mice Can be used in vaccine and gene therapy studies Can be used to study reversal of immunosuppression in GBMs (secretes immunosuppressive protein TGF-β which can be an immunotherapeutic target) Form spontaneous tumor	Relatively immunogenic compared to human GBM Not as well characterized and less commonly used compared to other models Homogeneous cell population
PDX	Human	GSCs and response to treatment and oHSV therapy High-throughput drug screening Retain primary tumor microenvironment and intra-tumoral heterogeneity Recapitulates histology and heterogeneity of human GBMs	Variability among lines Usually requires immunodeficient mice Can be difficult to establish and requires significant expertise

GBM, glioblastoma; GEMMs, genetically engineered mouse models; GSCs, glioma stem cells; oHSV, oncolytic herpes simplex virus; PDX, patient-derived xenograft; SMA, spontaneous murine astrocytoma.

### Genetically Induced Mouse Glioma Models

**FIGURE 2.**
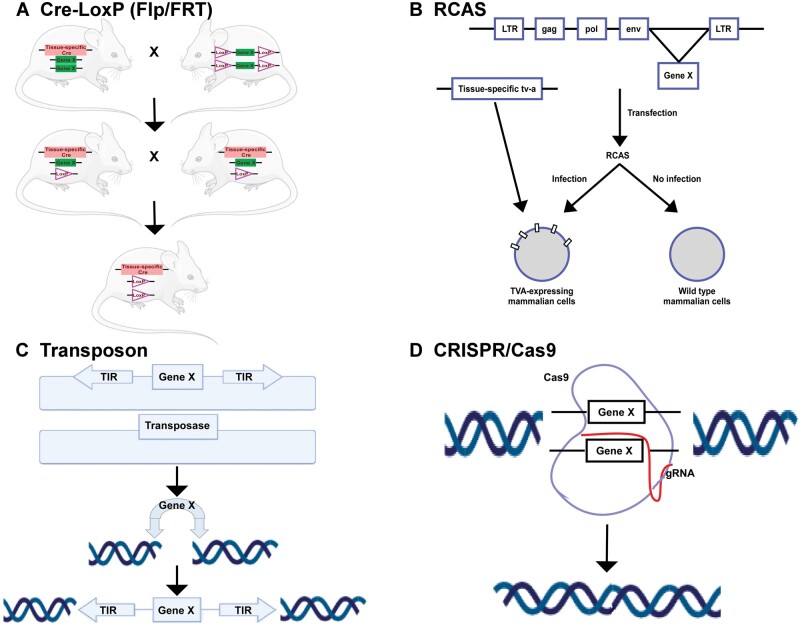
Illustration of various strategies employed to generate genetically engineered murine glioma models. **(A)** Cre-LoxP system. Tissue-specific Cre-expressing mice are crossed with transgenic mice engineered to have LoxP sites flanking a target gene. F1 generation then generates heterozygote mice that have floxed out one allele of the gene. Subsequent mating results in homozygous mice with tissue-specific loss of the target gene in both the alleles. **(B)** The RCAS-TVA system. RCAS retrovirus construct produced in DF1 chicken fibroblasts can exclusively enter mammalian cells expressing RCAS receptor, TVA. The neighboring TVA-negative mammalian cells cannot be infected by the RCAS virus. **(C)** Transposon system. Co-transfection of transposases with a genetic element containing the target gene (transposon) to be inserted, flanked by transposon-specific terminal inverted repeats (TIRs). Transposase excises the transposon resulting in integration of the target gene into a new target site in the mammalian genome. **(D)** CRISPR-Cas9 system. Cas9 enzyme mediate DNA incision at sequences directed by guide RNA (gRNA). Target genes are then edited by deleting or inserting new DNA at the cut position.

Advances in sequencing and genomic technologies have revolutionized our understanding of the changes and genetic alterations of GBMs (118, 119). This has led to the development of models with specific tumor driver mutations which are similar to human GBMs. These, GEM models, designed to harbor-specific mutations in a tissue-specific manner (120) have led to the development of unique mouse models that simulate human GBMs in their molecular alterations. These models facilitate the induction of a specific mutation in the choice cell of origin. This permits lineage-specific tracking during tumor development and it can be used to investigate TME interactions by permitting changes in specific genes of the TME. Different strategies used to generate these models have included different recombination, transposon, and virus-mediated gene transduction strategies. The molecular approaches that have been used to develop commonly used GEM models are shown in Figure 2.

### Conditional Cre/LoxP Site-Specific Recombination Driven Mouse Models

Cre-Lox is a site-specific recombinase technology derived from bacteriophage P1 based on the capability of the P1 bacteriophage cyclization recombination recombinase gene to produce recombination between 2 pairs of loxP sites (121). This strategy has been utilized for the creation of several insertions or deletions in the mouse genome as shown in Figure 2.

In 2000, Reilly et al (122) developed the first mouse model of astrocytomas with loss of 2 tumor suppressor genes- *Nf1* and *Trp53*. This resulted in the development of tumors ranging from low-grade astrocytoma to GBM. Later, Zhu et al (123) used Cre-Lox technology to generate mice deficient in *p53* and *NF1*−/− in GFAP-positive cells by cross-breeding *p53-*deficient mice harboring a conditional floxed allele of the NF1 tumor suppressor with mice expressing Cre under a GFAP promotor. These mice formed spontaneous astrocytomas with tumors that were similar to GBMs. In another study, cytomegalovirus immediate-early promoter driven loxP floxed red fluorescent protein-loxP, followed by *H-RasV12* was injected i.c. together with an *Akt*-expressing lentivirus into the hippocampus or subventricular zone of adult GFAP-Cre *Tp53* mice (124). In this model, GFAP-positive tumor cells expressed cre to drive recombination in a cell type-specific manner. Segregated 005 tumor cells from this model are highly tumorigenic and relatively non-immunogenic in C57BL/6 mice. These cells lack the expression of the co-stimulatory molecules CD80 and CD86 and the MHC-1 (125) and have been used in many studies (117, 126–131). Similarly, Nestin-cre has also been used to drive genetic alterations in nestin-positive cells (132).

### Flip/Flip Recognition Target Recombination System

Similar to Cre-lox, flip/flip recognition target (FLP/FRT)-directed site-specific recombination also has been used to generate GEM models. In this case, an altered FLP recombinase which initiated recombination between target FRT sites was used to generate gliomas in mice (133). This mouse model has facilitated additional manipulation of the mouse genome by Cre/LoxP, thereby conferring further understanding of the cellular and molecular mechanisms of gliomagenesis.

### Replication-Competent Avian Sarcoma Leukosis Virus Splice-Acceptor-Tumor Virus A-Mediated GEM Models

The avian sarcoma leukosis virus (ASLV) splice-acceptor system (RCAS) is a somatic gene transfer system that utilizes replication-competent RCAS vectors to target genetically engineered cells that express the cell surface receptor tumor virus A (TVA) for entry. Thus, tissue-specific expression of TVA can be exploited for cell-specific susceptibility to ASLV-derived RCAS virus. Mice expressing the TVA receptor under the regulation of a nestin promotor were infected with RCAS virus encoding for KrasG12D to create GBM-like brain tumors with Kras in nestin-positive cells (134). Furthermore, the transduction of RCAS-PDGFB into Ntv-a, Ink4a-ARF^−/−^ mice spontaneously developed brainstem gliomas (135). According to the current World Health Organization classification of tumors of the CNS (136), midline gliomas, previously termed diffuse intrinsic pontine glioma are characterized by H3 K27M mutations in the histone H3 gene *H3F3A* or *HIST1H3B*. Hoeman et al (137) established that transduction of RCAS-ACVR1 R206H with H3.1K27M into the brainstem of Ntv-a; Tp53fl/fl mice developed diffuse midline gliomas. However, the limitations of the RCAS model are the requirement for specific TVA-expressing mouse strains, and the capacity of the RCAS vector to carry a ∼2.5 kb insert. Likewise, several other viruses including adeno-associated viruses, adenoviruses, and lentiviruses have been employed for somatic gene transfer and production of GEM models. The advantage of these viruses over the RCAS virus is that entry is not restricted only to dividing cells.

### Transposon-Based Insertional Mutagenesis-Derived GEM Models

The identification of transposons or “mobile genetic elements” with the capability to change their location across the genome has facilitated the discovery of the transposon system driving inter-genomic migration. The transposition systems discovered to date include Sleeping Beauty (SB), PiggyBac (PB), Tol2, Frog Prince, Himar1, and passport (138). These transposon systems differ not only in their phylogenetic origin but also in the biological properties, including cargo size and DNA sequence preference for transposition. Among these transposon systems, SB and PB are the most effective and are widely used to develop GEM models as well as treatment for GBMs (139).

SB is a 2-part DNA transposon system that is used to insert tumor driver gene alterations in mouse models in several cancers including gliomas. This system is comprised of 2 important elements: SB transposase, an enzyme used for the mobilization of DNA, and the transposon containing the gene cassette that can translocate within the genome (140). The mechanism depends on a “cut-and-paste” system in which the transposase recognizes the inverted repeats of the transposon (IR/DR; inverted terminal repeat, direct repeat), excises it from the genome, and then that transposon can move to any location with a thymine-adenine dinucleotide region (∼200 million potential sites in mammalian genome) elsewhere in the genome (141). SB28 is a genetically engineered model induced by SB transposon-mediated intraventricular transfection of NRAS (neuroblastoma ras viral oncogene homolog), PDGF, and short hairpin Tp53 in neonatal C57BL/6 mice (142). This model exhibits a less immunogenic phenotype and a lower number of the predicted MHC-binding neo-epitopes compared to the spontaneous astrocytoma model SMA-560 and the MCA-induced GL261 model. Therefore, SB28 is resistant to immune checkpoint blockade (ICB) therapy (143, 144), while SMA-560 and GL261 models are susceptible to ICBs (145–148), but ICB is of questionable efficacy in human GBMs (149). Koschmann et al examined the effect of loss of ATRX (Alpha Thalassemia/Mental Retardation Syndrome X-Linked), which is often concurrent with TP53 mutation in gliomas using the SB system. In this model, the addition of SB-mediated shATRX to shTp53 and NRAS (NP) model significantly reduced the survival of tumor-bearing mice and augmented genetic instability (150). PB is another transposon system isolated from the cabbage looper moth (*Trichoplusia ni*) and is an efficient alternative to SB. While the original PB transposase exhibited higher transposase activity than SB (151), subsequent efforts to improve transposon efficiency resulted in the generation of hyperactive PBase and SB100X, with better transposon efficiency (152).

Izsvák et al reported decreased transposition efficiency with increasing transposon length with SB transposase. With each kb increase in transposon length, the efficiency of transposition decreased by ∼30% (153), and the cargo size limitation in SB is ∼5 kb (154). Compared with SB, PB elements can possibly carry larger cargos up to 9.1 kb of a foreign sequence without significantly decreasing the integration efficiency (155). Another advantage of PB is that it sustains overproduction inhibition, allowing tolerance to its induced expression, making it a viable option for developing GEM models, as well as gene delivery treatment in vitro and in vivo. Chen et al (126) devised a GEM glioma model by in utero electroporation of the PB transposon housing HRasV12 and AKT with GLAST-PBase into radial glial progenitors. Histologically these tumors were diffusely infiltrative into contiguous normal brain and were composed of proliferative cells with necrotic foci.

### CRISPR-Cas9 System

As described above to this point, the goal has been to create GEM models expressing tumor drivers. However, the deletion of tumor suppressor genes using the techniques described above is a tedious and time-consuming process involving homologous recombination of embryonic stem cells. Clustered, regulatory, interspaced, and short palindromic repeats (CRISPRs) were first identified in *Escherichia* *coli* in 1987 (156). CRISPR-associated protein 9 (Cas9) is an enzyme that recognizes CRISPR sequences by using single-guide RNAs (sgRNAs) and induces double-stranded cleavage of specific strands of DNA. This technology can be modified to cleave DNA in a sequence-specific manner leading to precise gene editing by avoiding cumbersome recombination strategies. Development of the CRISPR/Cas9 system is considered the ultimate transition since polymerase chain reaction and has revolutionized the current gene-editing method, as was recognized by the awarding of the 2020 Nobel Prize in Chemistry to Drs. Jennifer Doudna and Emmanuelle Charpentier.

Until now, several GEM models have been developed by CRISPR-Cas9 technology. For example, Zuckermann et al (157) developed a mouse model for sonic hedgehog medulloblastoma by depleting the *Ptch1* gene. Furthermore, they produced gRNAs targeting *Nf1*, *Trp53*, and *Pten*, resulting in aggressive tumors resembling human GBMs. Cook et al used a somatic CRISPR/Cas9 approach to produce a chromosomal rearrangement. The resulting Bcan-Ntrk1 fusion created a potent driver for GBM-like tumors in an in vivo study (158). Yu et al (159) reported a mouse glioma model by in utero electroporation of CRISPR/Cas9 vectors targeting *Nf1*, *Trp53*, and *PTEN* with different variants of *Pik3ca* mutations. The CRISPR/Cas9 system is a powerful method for generating transgenic mice by virtue of its simplicity, cost-effectiveness, efficacy, and relatively low toxicity even at relatively high doses of Cas9 mRNA and sgRNA.

### Spontaneous Tumor-Derived Implantable Mouse Models

The formation of spontaneous mouse tumors by producing specific gene mutations characteristic of human tumors represents a major advance because the tumor mutations are predictable. The use of spontaneous tumor-forming mouse models in the context of brain tumor modeling is challenging, if not impossible, with low tumor penetrance, extended latency before tumors form, poor reproducibility, and the need for advanced in vivo imaging techniques. Until 1971, the initial attempts to develop a spontaneous mouse glioma model were unsuccessful despite the development of murine, canine, and feline primary brain tumor models (160). In 1971, Fraser (161) developed a spontaneous mouse astrocytoma (SMA) cell line from the inbred VM/Dk strain that lost tumorigenicity with successive in vitro passages over time. A decade later Bigner et al defined 5 distinct cell lines (P492, P496, P497, P540, and P560) from the intracranial passaged SMA and developed 5 cell lines with astrocytic features that retained their tumorigenicity. Of these, SMA-560 has been used most extensively to evaluate various immunotherapeutic approaches (162–164). The 4C8 cell line replicates a non-chemically induced transplantable tumor. Originally derived from MOCH-1 glial transgenic mice, these cells form highly cellular tumors upon implantation into the F1 generation of C57BL/6J (B6) female x DBA/2J (D2) male crosses; they are thus heterozygous for all strain-specific loci in their genome (165).

Transduction of AKT and H-Ras in GFAP-Cre Tp53^−/−^ mice utilizing lentivirus have been used to produce spontaneous tumors in mice. These tumors then were used to obtain primary tumor cell cultures leading to the isolation of the 005 cell line, which formed neurosphere-like structures and it retained self-renewal and tumorigenicity upon subsequent i.c. implantation into mice. Furthermore, tumors derived from i.c. implantation of these cells are now frequently used (124). Cells derived from genetic models with inactivation of *NF1* and heterozygous for *p53* with or without *PTEN* inactivation also have been used in various studies. However, it is important to recognize that these cells originated from a mixed genetic background of C57BL/6/Sv129, and B6/CBA mice. Therefore, they are not syngeneic with any mouse inbred strain. RNAseq analysis of the end-stage tumors revealed that only the Mut3 tumors had an expression profile that was different from CT2A, GL261, or 005 mouse glioma cells. Furthermore, differentially expressed gene ontology pathways related to immune signaling were more greatly expressed in the GL261 and 005 than compared to Mut3- and CT2A-derived tumors (117).

### Human Glioma Xenograft Models

Xenografts of patient-derived glioma cells implanted into immunodeficient mice are the most widely used mouse models to investigate GBM growth and their response to therapeutics. Among these, the U87, which was derived from a patient with a malignant glioma, has been the most widely utilized model. After years in tissue culture, U87 cells have undergone clonal selection resulting in cells that grow rapidly in vitro and in vivo. Stable transduction of these cells with luciferase allows tracking of tumor growth in live animals by means of bioluminescence imaging (166, 167). Developed by Ponten and McIntyre at the University of Uppsala in 1966 (168), this cell line was widely distributed among brain tumor researchers and eventually was deposited in the ATCC. However, decades later, short tandem repeat sequence profiling of the cell line revealed that the U87 cells that were broadly used and distributed by ATCC did not match the patient tumor profile from whom these cells purportedly were derived. However, transcriptional profiling of the U87 cell line from the ATCC confirmed the CNS origin as a malignant glioma (MG), hence the original designation as U87MG. There is, however, some uncertainty to the gender of the patient of origin (169). These established human cell lines grow when implanted i.c. to immunocompromised mice and rats, and provide models with predictable and reliable tumor growth. However, they fail to display the histological hallmarks of GBMs such as invasive pattern of growth, necrotic foci within tumors, and microvascular proliferation. Based on this, xenograft models have transitioned to primary patient-derived stem cell-like cells maintained in vitro under conditions that do not permit differentiation or passage in mice as xenografts (170).

The shortcomings of the tumors derived from these traditional glioma cell lines, which have been maintained in serum-containing media, to fully reflect the genetic and histologic features of human GBMs has led to the use of PDX or genetically engineered glioma models (171). These models retain both the genetic and histologic features of human GBMs in immunocompromised animals. The most extensively used resource for GBM PDX cell lines is the Brain Tumor PDX National Resource at the Mayo Clinic. This center has characterized and annotated a series of brain tumor PDX models at a multi-omics level comparable to those provided for primary patient tumors by The Cancer Genome Atlas (TCGA). Unlike the traditional serum-grown cell lines such as U87, these models more closely resemble the histologic characteristics of the human tumors and express known GBM molecular markers such as mutant *TERT*, *EGFR* amplification, *PTEN* loss of heterozygosity, and mutant *IDH1*. Gene expression studies have revealed that these models also often reflect different molecular subtypes identified in GBM patients. Similar to human GBMs, the models include both methyl guanine methyl transferase (MGMT) methylated and unmethylated tumors and MGMT expression-mediated in vitro and in vivo resistance to TMZ (7). Integrated molecular profiling of patient-derived models has revealed that these tumors recapitulate most of the tumor-driving mutations reported in GBMs (172). While these models provide an excellent approach for accessing tumor-specific responses, the use of immunodeficient animals is a significant limitation for the evaluation of therapeutic responses. Furthermore, the requirement that these tumors must be passaged in mice also makes the models cumbersome and expensive.

### Humanized Mouse Models

The development of PDX models has improved the reliability of tumor models by providing models that more closely mimic the pathology of human tumors (171). They provide the inter- and intra-tumoral heterogeneity and also enable phenocopy sensitivity or resistance to targeted therapies. However, having been developed in immunodeficient animals, PDX models are limited to chemotherapeutic interventions. Immunotherapeutic options would require an intact immune system. Therefore, creating a mouse with human immune system (a “humanized mouse”) will be a better vehicle to facilitate the investigation of the human tumor-human immune cell interaction, and for testing anticancer immune responses for specific immunotherapeutic interventions.

The possibility of engraftment of human immune cells into immunodeficient mice initially was described in nude mice devoid of T cells (athymic nude mice) (173). However, there is a direct correlation between the engraftment and the degree of immunodeficiency of the host animals (174). Despite the availability of diverse types of immunodeficient mouse models, they fail to grow primary cancer cells or tissues. The development of severe combined immunodeficiency (SCID) mice, which are deficient in both T and B lymphocytes, and crossing them with the non-obese diabetic mice with deficiencies in natural killer (NK) cells, macrophages, and the complement system have been a major step forward (175, 176). Further improvements have led to the development of NOD/SCID mice with the elimination of the gamma chain of the IL-2 receptor (NSG), the loss of mouse NK cells, and non-functional IL-2, IL-4, IL-7, IL-9, IL-15, and IL-21 cytokines. The severe immunodeficiency of these modified animals has led to engraftment of almost all types cancers (177, 178). These mice have lymphocytes of human origin and chimeric hematopoietic cells, partially of human and partially of mouse origin. Ultimate success occurred when Takenaka et al (179) identified the signal-regulatory protein alpha (Sirpa) that strongly interacts with human CD47. The binding of Sirpa to CD47 results in inhibitory signals for macrophages thereby preventing their phagocytic activity and presumably making the strain permissive to a higher level of engraftment following the implantation of human cells.

A major component of the humanized mouse models is the type of cells used for engraftment. The first reported model focused on reconstitution using human peripheral blood mononuclear cells (PBMCs) in a SCID background. Although this model provided efficient T-cell engraftment, which resulted in mice with low levels of PBMC with human stem cell (HSC) engraftment, it lacked human B cells and myeloid-derived cells (180, 181). Another limitation was the development of lethal graft-versus-host-disease (GVHD) at approximately 4–6 weeks following engraftment (182, 183). Based on the robust GVHD response, which limited the survival time of the mice and thereby confounded the results, an alternative approach was used with the engraftment of CD34-positive HSC (184, 185). This approach allowed the development of human T cells with the selection of human MHC molecules thereby limiting the GVHD response. CD34-positive HSC models are predominantly limited by the degree of engraftment where the recipient mouse selection (age, sex, and strain), source of CD34-positive cells, and route by which engraftment occurs are the determining factors for the success of engraftment (177, 178, 186, 187). The success of the CD34-positive model is also contingent upon the depletion of the mouse HSC compartment with sub-lethal gamma-irradiation to facilitate human HSC engraftment. Optimization of these variables allows engraftment of human cells to approach >90% in many immune compartments, including the peripheral blood lymphocytes, spleen, thymus, and gut (184). However, the lack of cross-reactivity between mouse and human cytokines in all of these models provides a hostile environment for the maturation of the injected human PBMC or CD34-positive stem cells and thereby hinders an efficient repopulation into a mature immune system. Repeated injections of human cytokines or genes encoding plasmids in mice could possibly mitigate this limitation but can be challenging. The use of transgenic mice that express human rather than mouse cytokines ensured appropriate dosage and tissue specificity and permitted improved maturation and repopulation with mature human T cells, macrophages, and NK cells. Although these models are not allogeneic, they allow for the use of defined MHC types, with reconstitution allowing for monitoring and tracking of epitope-specific responses. As summarized in a recent review, these models are an improvement over currently used models, however, the high costs of these transgenic mice limits their extensive use (188).

### The Utility of Brain Tumor Models in the Development of New Therapeutic Approaches

Perhaps the first and one of the best examples of how rodent brain tumor models have led to clinical advances in the treatment of human brain tumors was the introduction of 1,3-Bis(2-chlorethyl)-1 nitrosourea (BCNU) for the treatment of patients with a variety of human cancers, including brain tumors of various histopathologic types (189). This important advance was based on studies carried out by Schabel et al (190) using a very unconventional brain tumor model, the i.c. implantation of L1210 leukemia cells into BDFl mice. BCNU, administered subcutaneously, was the most effective among a panel of nitrosoureas in increasing the lifespan of L1210 tumor-bearing mice. Based on this important observation, Walker et al administered BCNU i.v. to treat a total of 25 patients with astrocytomas and GBMs and a smaller number of patients with other types of primary brain tumors. Significant objective responses were seen in all groups of patients which quickly led to the widespread clinical use of BCNU to treat patients with brain tumors (191).

Even more important than BCNU were experimental brain tumor studies that led to the introduction of TMZ for the treatment of GBMs. Friedman et al (192) evaluated TMZ for the treatment of both s.c. and i.c. human xenografts derived from adult high-grade gliomas and 2 childhood tumors, an ependymoma and a medulloblastoma. The growth delays produced by TMZ in these xenografts transplanted into athymic (“nude”) BALB/c mice were highly significant, as determined by prolongation of median survival times. These studies suggested that TMZ might be active for the treatment of a broad range of CNS tumors and subsequently was followed by a clinical trial by Friedman et al (193). This was confirmed by a European Organisation for Research and Treatment of Cancer (EORTC) trial conducted by Stupp et al (194) and reported in a landmark paper that established the clinical efficacy of TMZ in combination external beam photon irradiation (6, 193).

Studies closely related to the BNCT research of one of us (R.F.B.), were carried out by Coderre et al (195) at the Brookhaven National Laboratory (BNL) on the potential use of boronophenylalanine (BPA) as a boron delivery agent for BNCT of patients with GBMs. BPA had been used by Mishima et al (196) for BNCT of patients with cutaneous melanomas. Coderre et al (195) first established that BPA potentially could be used as a boron delivery agent for tumors other than melanomas. Subsequently, he and his coworkers carried out studies in Fischer rats bearing i.c. implants of the 9L gliosarcoma that had received BPA as a boron delivery agent followed by BNCT. These studies established the therapeutic efficacy of BPA for the treatment of a brain tumor, and this quickly led to a clinical trial at BNL that provided some evidence of therapeutic efficacy for the treatment of patients with recurrent GBMs. Shortly thereafter, this led to the widespread clinical use of BPA as the best currently available boron delivery agent for BNCT to treat patients, not only with high-grade gliomas (197, 198) but also those with recurrent head and neck tumors (199, 200) and genital malignancies (201).

A third example of the utility of a rat brain tumor model is the C6 glioma, which led to the use of alternating electric fields or, as this modality is now known, “tumor treating fields” (TTF) to treat patients with recurrent GBMs (202). Initially, extensive animal studies were carried out by Kirson et al (203) using the F98 rat glioma. In vivo MRI studies demonstrated that alternating electric fields applied over the skulls of C6 glioma-bearing rats resulted in significant reductions in tumor growth as compared to that seen in untreated controls. This outcome led to the initiation of clinical studies using TTF for patients with recurrent GBMs (204).

Finally, we would like to briefly describe the clinical use of oncolytic HSV that recently has been reviewed by Nguyen and Saha (205). The potential use of oncolytic HSV for the treatment of human gliomas first was reported by Martuza et al (206). They used intratumoral injection of an HSV mutant *dlsp+k* in a xenograft model to treat nude mice bearing i.c. implants of the U87 glioma. A significant, dose-dependent increase in survival times was observed in 2 of 7 mice that had received 10^5^ pfu of dlsp+k. Among all of the oncolytic HSVs being evaluated, one “T-VEC” with an oncolytic HSV tolimogene laherparepvec, has been evaluated in a Phase I trial for treatment of patients with a variety of tumors. As recently reviewed by Koch et al (207) and Lu et al (208), 10 unique viral vectors are being evaluated in Phase I trials for the treatment of patients with GBMs. The most common of these were adenovirus vectors, but their therapeutic efficacy is yet to be determined. In summary, rat and mouse brain tumor models have played a significant role in the development of new therapeutic approaches to treat brain tumors, but the full potential of any of these has yet to be realized.

## CONCLUSIONS AND FUTURE PROSPECTS

We conclude by comparing the immunogenicity of human GBMs with that of the 3 rat brain tumors models that we have discussed, the C6, 9L, and F98 gliomas, and the one transplantable mouse tumor, the GL261. In contrast, the intrinsic immunogenicity of human GBMs is uncertain, and this in part has been attributed to the highly immunosuppressive TME (209). Therefore, caution must be taken when relating the efficacy and relevance of various therapeutic approaches in these rodent models to human GBMs. This was clearly demonstrated by Smilowitz et al (84) who observed that Fischer rats bearing 9L tumors and were treated by BNCT developed a strong immune response directed against further i.c. challenge of the tumor. Although significant clinical advances have been made based on studies carried out using rodent brain tumor models, one must take into account that the TME of rodent brain tumors does not seem to be immunosuppressive. Therefore, the encouraging therapeutic responses obtained with these models must be interpreted with caution when extrapolating them to human GBMs.

Rat and mouse brain tumor models have provided a wealth of information on the biology and biochemistry of brain tumors and elucidating events associated with the development, progression and treatment of brain tumors. However, it is essential to recognize the limitations of each of the models that we have described in this review. Depending on the nature of the study, it is important that the appropriate model be selected. This review has summarized the 3 most frequently used rat models and a variety of mouse brain tumor models that have been used in neuro-oncology research. Since some of these models were based on carcinogen-induced tumors, they failed to exhibit the genetic alterations seen in human GBMs. Current deep genome sequencing and single-cell technologies have revealed the multiple genetic alterations involved and molecular changes associated with brain tumor development.

The advent of GEM models with fast and reliable techniques to genetically engineer cells in a tissue- and cell-specific manner have increased the utility of mouse models to elucidate the molecular drivers that lead to oncogenesis. Mice with specific genetic alterations have proven useful to evaluate precisely designed targeted therapies to treat a cell population expressing a specific molecular target. There is, however, a paucity of genetically engineered rat brain tumor models. As described in our discussion of the F98 glioma, Barth et al (101) have produced and carried out extensive studies with an EGFR and EGFR_VIII_ expressing cell lines, Bryant et al (115) have produced a luciferase expressing cell line of the F98 glioma, and Connolly et al (210) have described the production of genetically engineered PDGF-driven tumor initiation and progression in tv-a transgenic Sprague Dawley rats that replicate key features of human gliomas. This was used to study glial brain tumors driven by 2 well-defined oncogenic gene transformations, PDGFA overexpression and p53 depletion using MRI and MR spectroscopy. However, these models do not replace the need for testing human tumors, something made especially important for species-specific biotherapies such as the use of oncolytic viruses and monoclonal antibodies that target key drivers of tumor progression. These therapeutic approaches mostly have been limited to immunodeficient mice that can accept human tumor xenografts.

While rodent brain tumor models have contributed to basic tumor biology and drug development, sadly, to date, improvements in the treatment of GBMs have only resulted in relatively modest increases in MST and barring an unexpected breakthrough, their ultimate cure is still a far-off goal. The reasons for this include limited penetration of the BBB by both low and especially high molecular weight therapeutic agents, development of drug resistance, and the cellular heterogeneity of GBMs. However, the rodent models have provided a wealth of information on the potential toxicity and efficacy of various therapeutic agents.

The development of cell lines, which were derived from human brain tumors, began decades ago. Although they failed to replicate the cellular and molecular heterogeneity observed in human GBM, this has resulted in a number of them that reproducibly produce tumors. The development of primary, patient-derived neurospheres or cells, which routinely can be passaged through mice as PDX, has been a major advance. However, they still are limited to use in immunodeficient mice. The touchstone for the study of human tumors is an intact TME in “humanized” mouse models that can accept human tumors and develop a mature human immune system. While these are cutting edge model systems, they are very costly and therefore are of limited utility. Further development of cost-effective and possibly isogenic humanized rodent models is a prerequisite to be able to precisely evaluate the safety and efficacy of the treatment modalities in preclinical studies. These should facilitate translation of a variety of therapeutic approaches that ultimately will result in cures for what has been the most refractory of all human cancers.

## Notes

Upasana Sahu, Rolf F. Barth, and Yoshihiro Otani contributed equally to this review.

BK received support from the National Cancer Institute, PO1 CA163205, and National Institute of Neurological Disorders and Stroke, R61 NS112410 and RO1 NS127473. RFB received support from the National Cancer Institute, 1RO1 CA098945-01, 1RO1 CA129326, and 1RO1 CA127935, Voices Against Brain Cancer, the Musella Foundation, and the Kevin Mullin Memorial Fund for Brain Tumor Research. RFB gratefully thanks David Carpenter for outstanding assistance in the preparation of this review.
